# Blood culture positivity and its clinical determinants among hospitalized male patients with newly diagnosed, laboratory-confirmed brucellosis: a hospital-based retrospective study

**DOI:** 10.3389/fmicb.2026.1754087

**Published:** 2026-02-04

**Authors:** Xue Han, Ruiqing Zhang, Bianxia Xu, Weijie Zhang, Fang Wang, Beibei Yuan, Quanlong Ma, Jing Ma, Zhenhua Zhu, Xiaofang Shan, Ye Liu

**Affiliations:** Department of Infectious Disease, The Fourth People’s Hospital of Ningxia Hui Autonomous Region, Yinchuan, Ningxia, China

**Keywords:** blood culture, brucellosis, clinical diagnosis, predictive factors, retrospective study

## Abstract

**Background and objective:**

Brucellosis is a common zoonotic disease worldwide. Because its symptoms are non-specific and laboratory findings vary, diagnosis remains challenging. Blood culture is still the gold standard for confirmation, but its yield is often limited. This study aimed to estimate blood-culture positivity among hospitalized male patients with newly diagnosed brucellosis and to identify clinical and laboratory factors independently associated with culture positivity within confirmed cases.

**Methods:**

We conducted a hospital-based retrospective study of 1,188 hospitalized men with newly diagnosed brucellosis admitted to a tertiary infectious-disease hospital in China between 2021 and 2023. Demographics, clinical manifestations, laboratory parameters, and blood-culture results were collected. Multivariate logistic regression was used to identify independent predictors of a positive blood culture.

**Results:**

The overall blood-culture positivity rate was 30.9%. Univariate analysis indicated that disease stage, fatigue, fever, anorexia, splenomegaly, arthritis, paraspinal abscess, joint effusion, platelet count (PLT), alanine aminotransferase (ALT), aspartate aminotransferase (AST), gamma-glutamyl transferase (GGT), albumin (ALB), total bilirubin, lactate dehydrogenase (LDH), serum agglutination test (SAT), procalcitonin (PCT), and erythrocyte sedimentation rate (ESR) were associated with culture positivity. After multivariate adjustment, LDH, SAT, PCT, and ESR emerged as independent predictors of a positive blood culture, with PCT showing the strongest predictive value. Higher levels of LDH, SAT, PCT and ESR were independently associated with higher positive rates of blood cultures.

**Conclusion:**

In this hospital-based retrospective cohort of confirmed brucellosis, LDH, SAT titer, PCT, and ESR were independently associated with blood-culture positivity. These routinely available parameters may help risk-stratify bacteremic likelihood and support culture-related decision-making (e.g., prioritizing sampling and considering repeat cultures when clinically warranted) among SAT-positive (or otherwise confirmed) patients, but they should not be interpreted as standalone markers for diagnosing *Brucella* bacteremia without microbiological confirmation.

## Introduction

Brucellosis is a global zoonotic infectious disease caused by the genus Brucella, which mainly affects humans and animals, especially those who are closely in contact with livestock, such as agricultural and pastoral workers (Dean et al., 2012). The disease is mainly transmitted through direct contact with infected animals or their body fluids, or through the consumption of raw dairy products or unpasteurized dairy products from infected animals (such as milk and cheese) (Khurana et al., 2021; Fritz et al., 2021). In recent years, the number of human brucellosis cases worldwide has increased sharply, with approximately 2.1 million new cases per year (Laine et al., 2023). According to Chinese surveillance data, the overall incidence rate of brucellosis has risen from 0.92 cases per 100,000 people in 2004 to 4.2 cases per 100,000 people in 2014, and reached 4.95 cases per 100,000 people in 2021 (Lu et al., 2024).

The clinical manifestations of brucellosis are complex and diverse, including headache, recurrent fever, migratory joint pain, muscle pain, weakness, anorexia, fatigue, malaise, sweating, vomiting, diarrhea, abdominal pain, and even abortion (Głowacka et al., 2018). The complexity of its clinical manifestations often leads to misdiagnosis. If not treated in time, the condition may become chronic, such as arthritis, heart disease, and neurological damage, thereby increasing the risk of disability and bringing a huge economic burden to patients, families, and society (Ulu-Kilic et al., 2013; Pappas et al., 2005). Epididymo-orchitis is the most common urogenital complication of brucellosis, with about 2–20% of male patients developing this complication after being infected with Brucella (Savasci et al., 2014; Salmeron et al., 1998; Gozdas and Bal, 2020). The disease usually occurs in young people, with an average patient age of 30–40 years, and has a serious impact on the reproductive health of young men (Savasci et al., 2014; Roushan et al., 2009).

Blood culture is the “gold standard” for confirming brucellosis, but its results are prone to false negatives due to various factors (Di Bonaventura et al., 2021; Yagupsky et al., 2019; Kara and Cayir, 2019). However, culturing *Brucella* spp. is often time-consuming and operationally demanding. The organism is slow-growing and may require prolonged incubation and, in some settings, terminal and/or blind subcultures, which increase hands-on laboratory workload and delay confirmation (Di Bonaventura et al., 2021; Freire et al., 2024; ASM, 2025). In addition, handling *Brucella* requires strict biosafety precautions and trained personnel, posing further challenges for routine diagnosis in highly endemic areas (Di Bonaventura et al., 2021; ASM, 2025). Previous studies have shown that the patient’s age, duration of symptoms, acute or chronic course of disease, focal disease, primary infection or relapse, and antibiotic use can all affect the positivity rate of Brucella blood culture (Di Bonaventura et al., 2021). Many studies have investigated the factors affecting the results of brucellosis blood culture. Two studies on pediatric patients with brucellosis showed that young age, fever, arthralgia, hepatosplenomegaly, high C-reactive protein (CRP) levels, and high liver function enzyme levels are key factors determining positive blood culture (Özdem et al., 2022; Apa et al., 2013). Another study from Turkey found that shorter duration of symptoms, no personal or family history of brucellosis, and higher levels of white blood cells, CRP, and ferritin are associated with positive blood culture (Kara and Cayir, 2019). However, a study on patients in the intensive care unit (ICU) showed that body temperature, neutrophil percentage, lymphocyte count, platelet count, CRP level, gender, and age have no significant impact on the positivity rate of blood culture (Wu et al., 2016). Although many studies have explored the factors affecting the positivity rate of blood culture, evidence focusing specifically on first-diagnosed chronic brucellosis—particularly among hospitalized male patients—remains limited, and whether routine clinical and laboratory features can assist culture-related decision-making in this subgroup has not been clearly addressed.

Accordingly, this study aimed to determine the clinical and laboratory determinants of blood culture positivity in a cohort of hospitalized male patients with first-diagnosed brucellosis in Ningxia, China—a high-burden endemic region. Using a hospital-based retrospective design, we analyzed a comprehensive cohort of patients with newly diagnosed, hospitalized brucellosis and compared the clinical and laboratory profiles of those with positive versus negative blood cultures. Our primary aim was to identify readily available clinical and laboratory predictors of culture positivity within confirmed cases, to support risk stratification and optimize culture-related decision-making (e.g., prioritizing sampling and considering repeat cultures when clinically warranted), rather than to propose standalone diagnostic markers.

## Materials and methods

### Study design and participants

This retrospective observational study was conducted from January 2021 to December 2023 at a tertiary infectious-disease hospital in China. To minimize selection bias, we enrolled consecutive eligible hospitalized male patients with a first diagnosis of chronic brucellosis during the study period, based on predefined inclusion and exclusion criteria. Inclusion criteria were: (1) a first-time diagnosis of brucellosis based on a positive serum agglutination test (SAT) or a positive blood culture for Brucella species; (2) complete clinical, laboratory, and epidemiological data available; and (3) age ≥ 16 years. Patients with incomplete records or an uncertain diagnosis were excluded.

This study complies with the Declaration of Helsinki. The study protocol was approved by the Ethics Committee of the Fourth People’s Hospital of Ningxia Hui Autonomous Region (IRB202201). Informed consent was waived due to the retrospective nature of the study and anonymization of all patient data.

### Data collection

Data were collected through a structured clinical questionnaire administered during hospitalization, with supplementary information extracted from the hospital’s electronic medical record system. Demographic and epidemiologic variables captured included age, educational level and body-mass index (BMI). Clinical characteristics recorded the presence or absence of fever, sweating, fatigue, arthralgia, spinal symptoms, splenomegaly and lymph-node enlargement.

Laboratory test data include blood culture results for Brucella species identification (e.g., *B. melitensis*, *B. abortus*), along with routine hematology parameters such as white blood cell count and platelet count. Immunological assessments cover procalcitonin (PCT) levels, erythrocyte sedimentation rate (ESR), and cytokine concentrations (including interleukin-6 IL-6). Liver function tests encompass alanine aminotransferase (ALT), aspartate aminotransferase (AST), *γ*-glutamyl transpeptidase (GGT), total bilirubin (TBIL), and serum albumin levels.

Imaging studies comprised spinal magnetic resonance imaging (MRI) to detect arthritis, spondylitis and paraspinal abscesses, as well as abdominal ultrasonography to evaluate joint effusion and related findings.

### Microbiological diagnosis

Blood samples were collected using aseptic techniques and inoculated into automated blood culture systems. Positive cultures were further subtyped based on phenotypic characteristics. The SAT was performed simultaneously as part of routine diagnostic procedures. A SAT titer ≥1:160 was considered positive, consistent with previous guidelines.

### Outcome measures

The primary outcome was blood culture positivity. Secondary outcomes included identifying factors associated with culture positivity among confirmed cases, including demographic characteristics, clinical symptoms, and laboratory parameters.

### Statistical analysis

Continuous variables were expressed as means ± standard deviations (SD) or medians with interquartile ranges (IQR) depending on the distribution. Categorical variables were presented as frequencies and percentages. Comparisons between blood culture-positive and blood culture-negative groups were performed using Student’s *t*-test or Mann–Whitney U test for continuous variables, and Chi-square test or Fisher’s exact test for categorical variables, as appropriate. Multivariate logistic regression analysis was used to identify independent factors associated with blood culture positivity. Variables with a *p*-value <0.001 in univariate analyses were included in the multivariate model. Adjusted odds ratios (OR) and 95% confidence intervals (CI) were reported. All statistical analyses were performed using SPSS version 25.0 (IBM Corp., Armonk, NY, USA). A two-sided *p*-value <0.05 was considered statistically significant.

## Results

### Baseline characteristics

Table 1 summarizes the baseline characteristics of the study population. A total of 1,188 male hospitalized patients diagnosed with brucellosis were included in this study. The average age was 48.39 years. 367 (30.9%) had positive blood cultures for brucella.

**Table 1:** Baseline characteristics of the study population.

Feature	Total (*n* = 1,188)
Age, years, Mean (SD)	48.39 (14.40)
Education level	
Illiterate, *n* (%)	160 (13.5)
Primary school, *n* (%)	374 (31.5)
Junior high school, *n* (%)	296 (24.9)
High school/Technical secondary school, *n* (%)	302 (25.4)
College/University, *n* (%)	43 (3.6)
Graduate and above, *n* (%)	13 (1.1)
BMI, kg/m^2^, Mean (SD)	23.02 (3.47)
Fatigue, *n* (%)	419 (35.3)
Arthralgia, *n* (%)	537 (45.2)
Hidrosis, *n* (%)	66 (5.6)
Myalgia, *n* (%)	22 (1.9)
Fever, *n* (%)	350 (29.5)
Lumbago, *n* (%)	325 (27.4)
Anorexia, *n* (%)	59 (5.0)
Splenomegaly, *n* (%)	271 (22.8)
Testicular swelling and pain, *n* (%)	37 (3.1)
Arthritis, *n* (%)	1,059 (89.1)
Rachitis, *n* (%)	444 (37.4)
Paravertebral abscess, *n* (%)	92 (7.7)
Intraspinal abscess, *n* (%)	46 (3.9)
Hydrops articuli, *n* (%)	879 (74.0)
Lymphadenopathy, *n* (%)	78 (6.6)
Temperature, °C, Mean (SD)	37.24 (0.89)
PLT, ×10^9^/L, Mean (SD)	212.32 (64.21)
ALT, U/L, Mean (SD)	25.26 (27.10)
AST, U/L, Mean (SD)	27.69 (34.27)
GGT, U/L, Mean (SD)	51.60 (62.52)
ALB, g/L, Mean (SD)	34.72 (4.28)
WBC, ×10^9^/L, Mean (SD)	5.09 (1.99)
Lymphocytes, ×10^9^/L, Mean (SD)	1.66 (0.65)
Total bilirubin, μmol/L, Mean (SD)	11.67 (9.21)
LDH, U/L, Mean (SD)	205.72 (101.07)
SAT, IU/mL, Mean (SD)	423.92 (274.71)
Amylase, U/L, Mean (SD)	71.10 (25.90)
PCT, ng/dL, Mean (SD)	9.44 (8.44)
IL-6, pg/mL, Mean (SD)	13.76 (25.45)
ESR, mm/h, Mean (SD)	23.81 (22.52)
Blood culture positive, *n* (%)	367 (30.9)

### Results of univariate analysis comparison of blood culture positive brucellosis

Univariate analysis showed that disease stage, fatigue, fever, anorexia, splenomegaly, arthritis, paraspinal abscess, joint effusion, PLT, ALT, AST, GGT, ALB, TBIL, LDH, SAT, PCT, and ESR were all associated with blood-culture positivity. Specifically, acute stage, fatigue, anorexia, arthritis, absence of paraspinal abscess, splenomegaly, joint effusion, higher body temperature, ALT, AST, GGT, TBIL, LDH, SAT, PCT, and ESR, together with lower platelet count and ALB, were linked to a positive culture (all *p* < 0.05, Table 2).

**Table 2:** Results of univariate analysis comparison of blood culture positive brucellosis.

Feature	Blood culture negative (n = 821)	Blood culture positive (n = 367)	*p*-value
Age, years, Mean (SD)	48.31 (14.53)	48.57 (14.12)	0.768
Education Level			0.922
Illiterate, *n* (%)	106 (66.3)	54 (33.8)	
Primary school, *n* (%)	264 (70.6)	110 (29.4)	
Junior high school, *n* (%)	203 (68.6)	93 (31.4)	
High school/Technical secondary school, *n* (%)	208 (68.9)	94 (31.1)	
College/University, *n* (%)	30 (69.8)	13 (30.2)	
Graduate and above, *n* (%)	10 (76.9)	3 (23.1)	
BMI, kg/m^2^, Mean (SD)	22.86 (3.35)	23.10 (3.52)	0.272
Stage, Mean (SD)			<0.001
Acute stage	643 (64.9)	348 (35.1)	
Subacute stage	103 (87.3)	15 (12.7)	
Chronic phase	75 (96.2)	3 (3.8)	
Fatigue, *n* (%)	273 (65.2)	146 (34.8)	0.030
Arthralgia, *n* (%)	374 (69.6)	163 (30.4)	0.715
Hidrosis, *n* (%)	41 (62.1)	25 (37.9)	0.206
Myalgia, *n* (%)	17 (77.3)	5 (22.7)	0.403
Fever, *n* (%)	190 (54.3)	160 (45.7)	<0.001
Lumbago, *n* (%)	235 (72.3)	90 (27.7)	0.143
Anorexia, *n* (%)	32 (54.2)	27 (45.8)	0.011
Splenomegaly, *n* (%)	138 (50.9)	133 (49.1)	<0.001
Testicular swelling and pain, *n* (%)	23 (62.6)	14 (37.8)	0.353
Arthritis, *n* (%)	715 (67.5)	344 (32.5)	<0.001
Rachitis, *n* (%)	314 (70.7)	130 (29.3)	0.353
Paravertebral abscess, *n* (%)	73 (79.3)	19 (20.7)	0.027
Intraspinal abscess, *n* (%)	34 (73.9)	12 (26.1)	0.472
Hydrops articuli, *n* (%)	592 (67.3)	287 (32.7)	0.027
Lymphadenopathy, *n* (%)	50 (64.1)	28 (35.9)	0.322
Temperature, °C, Mean (SD)	37.09 (0.73)	37.58 (1.09)	<0.001
PLT, ×10^9^/L, Mean (SD)	215.24 (66.59)	205.24 (66.59)	0.012
ALT, U/L, Mean (SD)	21.72 (22.15)	33.16 (34.53)	<0.001
AST, U/L, Mean (SD)	22.33 (24.40)	39.67 (47.60)	<0.001
GGT, U/L, Mean (SD)	46.56 (54.77)	63.56 (75.83)	<0.001
ALB, g/L, Mean (SD)	35.53 (4.47)	33.67 (4.12)	<0.001
WBC, ×10^9^/L, Mean (SD)	5.10 (1.85)	5.06 (2.29)	0.781
Lymphocytes, Mean (SD)	1.63 (0.61)	1.71 (0.72)	0.056
Total bilirubin, μmol/L, Mean (SD)	11.26 (9.12)	12.59 (9.38)	0.022
LDH, U/L, Mean (SD)	262.54 (138.14)	179.51 (62.98)	<0.001
SAT, Mean (SD)	375.94 (269.63)	533.33 (254.43)	<0.001
Amylase, U/L, Mean (SD)	72.79 (26.65)	68.31 (24.53)	0.228
PCT, ng/dL, Mean (SD)	7.36 (7.23)	13.46 (9.16)	<0.001
IL-6, pg/mL, Mean (SD)	12.96 (28.72)	15.46 (16.50)	0.401
ESR, mm/h, Mean (SD)	21.18 (21.59)	29.73 (23.48)	<0.001

### Multivariate analysis results of factors affecting blood culture outcomes

In the univariate analysis, factors with *p* values <0.001 were identified and included in the multivariate analysis. After adjusting for staging, fever, splenomegaly, arthritis, ALT, ALB, LDH, AST, PCT, and ESR, the results showed significant correlations between LDH, SAT, PCT, ESR, and blood culture positivity. For each unit increase in LDH, the brucellosis positivity rate increased by 1% (OR = 1.01, 95% CI: 1.0007–1.014, *p* < 0.001). SAT showed a 0.1% (OR = 1.001, 95% CI: 1.001–1.002 increase, *p* < 0.001), while PCT was associated with a 4% increase in positivity risk (OR = 1.04, 95% CI:1.00–1.08, *p* = 0.029). ESR also contributed to a 2% (OR = 1.02, 95%CI: 1.01–1.03, *p* < 0.001) (Table 3).

**Table 3:** Results of multivariate analysis comparison of blood culture positive brucellosis.

Feature	Reference group	OR (95% CI)	*p*-value
Stage	Acute stage		
Subacute stage		0.18 (0.02, 1.37)	0.097
Chronic phase		0.53 (0.23, 1.25)	0.149
Fever	No	1.09 (0.69, 1.73)	0.709
Splenomegaly	No	1.54 (0.94, 2.53)	0.086
Arthritis	No	1.19 (0.54, 2.66)	0.667
ALT		1.01 (0.999, 1.01)	0.097
ALB		0.97 (0.92, 1.03)	0.320
LDH		1.01 (1.007, 1.014)	<0.001
SAT		1.001 (1.001, 1.002)	<0.001
PCT		1.04 (1.00, 1.08)	0.029
ESR		1.02 (1.01, 1.03)	<0.001

## Discussion

This study systematically evaluated the blood-culture positivity rate and its clinical-laboratory determinants among hospitalized brucellosis patients. The overall positivity rate was 30.9%, and multivariate modeling further identified LDH, SAT, PCT, and ESR as independent predictors of bacteremia. These findings underscore the marked clinical heterogeneity of brucellosis and indicate that integrating key symptoms with biochemical parameters can significantly improve early detection of bacteremia. For the first time, LDH, SAT, PCT, and ESR have been simultaneously established as novel independent predictors, providing new evidence-based insights for optimizing diagnostic algorithms and resource allocation in brucellosis.

Previous studies have reported the blood-culture positivity rate in brucellosis. According to the duration of clinical symptoms, cases are classified into acute (< 6 months) and chronic (> 6 months) stages (Jiang et al., 2020). Early investigations noted the protean manifestations of the disease: fever, arthralgia and fatigue were the most frequent, occurring in 23.33% (371/1590), 37.30% (593/1590) and 42.96% (683/1590) of patients, respectively, with 59.57% (221/371) exhibiting intermittent low-grade fever (Liu et al., 2023). In a cohort of 294 laboratory-confirmed brucellosis patients, only 40 (13.6%) had positive blood cultures, and the positivity rate in the acute phase was 46.8% (Konya et al., 2023). Another survey from Hulunbuir, Inner Mongolia, documented a yield of 14.9% (Liang et al., 2019). The present study found an overall blood-culture positivity of 30.9%, and arthralgia, fatigue and arthritis were commonly observed—findings that accord with earlier reports and corroborate the epidemiological and clinical features of human brucellosis.

The determinants of blood-culture positivity in brucellosis remain contentious. Previous work suggests that the yield is governed by two broad categories of factors (Shi et al., 2021). The first relates to the patient’s disease status—age, duration of symptoms, presence of systemic or focal infection, primary versus relapsing episode, and prior antibiotic exposure. The second concerns the culture methodology itself: total volume of blood sampled, number of culture sets, frequency of monitoring, sensitivity of the blood-culture system, incubation time, and the use of blind sub-cultures both during and at the end of incubation. Early in the disease, patients often have high-level serum bacteremia, giving higher culture positivity; as the illness progresses, bacteria are cleared or sequestered in tissues and organs, so blood concentrations fall and the yield declines (Kara and Cayir, 2019). Earlier reports showed that ALT, AST, TBIL, serum creatinine (SCr) and PLT are all significantly higher in the acute and sub-acute phases than in the chronic phase (Bai et al., 2023). In a cohort of 109 brucellosis patients, fever was the most common symptom (89.0%), followed by chills (52.3%), arthralgia (48.6%) and weight loss (30.3%) (Shi et al., 2021). The bacteremic group had significantly higher rates of fever (96.1%), arthralgia (58.8%), anorexia (35.3%), leukopenia (31.4%) and elevated AST (51.0%) than the non-bacteremic group. Our multivariate analysis identified LDH, SAT, PCT and ESR as independent predictors of a positive blood culture, underscoring the pivotal role and predictive value of systemic inflammation in the pathogenesis of brucellosis. By focusing on newly diagnosed, hospitalized patients, this study provides the first large-scale characterization of the epidemiology and clinical spectrum of brucellosis in China, offering novel insights into diagnosis and important evidence for therapeutic decision-making and prognostic assessment.

Notably, LDH, ESR, and PCT are non-specific inflammatory markers and may be influenced by a broad range of bacterial infections, inflammatory conditions, and other pathologies. Therefore, these parameters should not be interpreted as standalone tests for diagnosing brucellosis or *Brucella* bacteremia, nor do they replace microbiological confirmation. Importantly, the associations observed in our study were derived within a cohort of confirmed brucellosis patients, in whom SAT positivity (and/or other confirmatory evidence) established the clinical diagnosis prior to evaluating predictors of blood culture positivity. In this context, elevated LDH, ESR, and PCT may reflect a higher inflammatory burden and could be interpreted as adjunctive indicators of bacteremic likelihood among SAT-positive patients. From a practical perspective, these routinely available markers may assist early clinical decision-making in confirmed cases—for example, to prioritize obtaining blood cultures, ensure appropriate sampling volume/timing, and to consider repeat cultures when the initial culture is negative but clinical suspicion of bacteremia remains high. Nevertheless, given their limited specificity and the retrospective design, these markers should be applied cautiously and always interpreted alongside epidemiological exposure, clinical manifestations, and confirmatory testing strategies.

### Limitations

This study has several limitations that should be acknowledged. First, as a single-center retrospective study conducted in a specialized infectious disease hospital, there is a potential for selection bias, and the findings may not be generalizable to other populations, such as community-based or asymptomatic brucellosis cases. To mitigate selection bias, we implemented the following measures: (i) continuous enrollment of eligible hospitalized patients during the study period to reduce selective enrollment; (ii) application of predefined inclusion and exclusion criteria, excluding only patients with missing key data or uncertain diagnoses; (iii) analysis of determinants of blood culture positivity in confirmed brucellosis cases to minimize heterogeneity caused by misclassification; (iv) adjustment of clinically relevant covariates in multivariate models, with reported effect estimates including confidence intervals. Future multicenter, prospective studies are needed to confirm the external validity of our results. Second, although blood culture was used as the diagnostic reference standard, variability in collection techniques, timing of sampling, and culture conditions may have influenced the true positivity rate. Standardizing culture protocols and incorporating molecular diagnostic methods could help address this issue. Third, clinical symptoms such as fatigue, myalgia, and weight loss were based on patient-reported data and medical records, which may be subject to recall bias or incomplete documentation. Prospective studies using standardized symptom assessment tools would improve accuracy. Furthermore, CRP was not routinely tested in all patients during the study period, and thus CRP was not included in our analysis. This prevented direct comparison with previous studies that reported CRP as a determinant of positive blood culture for brucellosis. Additionally, this study only included male patients with first-time confirmed brucellosis and did not encompass female patients. This gender limitation implies that the study findings cannot be directly generalized to the female patient population. Given that the clinical manifestations, pathological mechanisms, and treatment responses of brucellosis may differ between male and female patients, future studies should consider enrolling female patients to comprehensively evaluate the impact of brucellosis on patients of different genders.

This study did not include reproductive function-related indicators such as “infertility” as research variables. Although we recognize that “infertility” may be a significant health issue in brucellosis patients, the primary objective of this study was to focus on the acute symptoms and short-term therapeutic outcomes of brucellosis. Therefore, reproductive function-related indicators were not incorporated as research variables. Future studies should consider evaluating “infertility” as an important outcome variable to gain a more comprehensive understanding of the long-term health impacts of brucellosis on patients. Finally, data on prior antibiotic exposure were not systematically collected, which may have led to underestimation of blood culture positivity, particularly in patients who received empirical treatment before admission. In this study, continuous monitoring of antibiotic exposure and its temporal relationship with procalcitonin (PCT) testing and blood culture sampling was not achieved. Due to the potential decline in PCT following effective antimicrobial therapy and its possible influence from prior empirical treatment, we were unable to evaluate or correct antibiotic-associated PCT kinetic changes, nor could we fully quantify its impact on culture positivity rates. Future research should include detailed information on pre-hospital antibiotic use to better assess its impact on culture outcomes.

## Conclusion

This study provides new insights into the clinical and laboratory factors associated with positive blood cultures among hospitalized patients with confirmed brucellosis. It highlights that LDH, SAT titer, PCT, and ESR are independently associated with blood culture positivity. Importantly, because LDH, ESR, and PCT are non-specific inflammatory markers that may also be altered in other bacterial infections or inflammatory conditions, they should be interpreted only within SAT-positive (or otherwise confirmed) brucellosis cases and should not be considered standalone indicators for diagnosing *Brucella* bacteremia. For patients, these findings underscore the importance of timely confirmatory testing and diagnosis, which can facilitate early treatment, delay disease progression, and prevent long-term complications. For clinicians, the results may support early decision-making in confirmed cases, such as prioritizing blood-culture sampling and considering repeat cultures when clinically warranted, thereby improving diagnostic efficiency while maintaining diagnostic rigor. From a societal perspective, evidence-based strategies that optimize confirmatory testing workflows in endemic areas could improve resource allocation and potentially reduce healthcare costs. Future prospective, multi-center studies are warranted to validate these associations and refine risk-stratified diagnostic pathways in diverse populations.

## Data Availability

The raw data supporting the conclusions of this article will be made available by the authors, without undue reservation.
